# homeSound: Real-Time Audio Event Detection Based on High Performance Computing for Behaviour and Surveillance Remote Monitoring

**DOI:** 10.3390/s17040854

**Published:** 2017-04-13

**Authors:** Rosa Ma Alsina-Pagès, Joan Navarro, Francesc Alías, Marcos Hervás

**Affiliations:** 1 GTM—Grup de recerca en Tecnologies Mèdia, La Salle—Universitat Ramon Llull, C/Quatre Camins, 30, 08022 Barcelona, Catalonia, Spain; falias@salleurl.edu (F.A.); mhervas@salleurl.edu (M.H.); 2 GRITS—Grup de Recerca en Internet Technologies & Storage, La Salle—Universitat Ramon Llull, C/Quatre Camins, 30, 08022 Barcelona, Catalonia, Spain; jnavarro@salleurl.edu

**Keywords:** Ambient Assisted Living, Acoustic Sensor Network, machine hearing, audio feature extraction, data mining, graphics processor unit, behaviour monitoring, surveillance

## Abstract

The consistent growth in human life expectancy during the recent years has driven governments and private organizations to increase the efforts in caring for the eldest segment of the population. These institutions have built hospitals and retirement homes that have been rapidly overfilled, making their associated maintenance and operating costs prohibitive. The latest advances in technology and communications envisage new ways to monitor those people with special needs at their own home, increasing their quality of life in a cost-affordable way. The purpose of this paper is to present an Ambient Assisted Living (AAL) platform able to analyze, identify, and detect specific acoustic events happening in daily life environments, which enables the medic staff to remotely track the status of every patient in real-time. Additionally, this tele-care proposal is validated through a proof-of-concept experiment that takes benefit of the capabilities of the NVIDIA Graphical Processing Unit running on a Jetson TK1 board to locally detect acoustic events. Conducted experiments demonstrate the feasibility of this approach by reaching an overall accuracy of 82% when identifying a set of 14 indoor environment events related to the domestic surveillance and patients’ behaviour monitoring field. Obtained results encourage practitioners to keep working in this direction, and enable health care providers to remotely track the status of their patients in real-time with non-invasive methods.

## 1. Introduction

The average life expectancy of the world’s population has steadily increased during the last decades [1]. In addition, it is expected to keep growing during the next century [2]. As a consequence, the number of patients, especially the eldest ones who require health care assistance in medical facilities, has increased accordingly [3,4,5]. To address this situation, health care providers have increased the number of hospitals and medical facilities as well as the number of health care professionals committed to attend an ever-rising figure of patients. However, this situation has opened several concerns in terms of both operational expenses and users experience. One the one hand, the growth of the health care system has become difficult to sustain with public funding in several countries [6]. On the other hand, moving patients from home to the medical facilities for a routinely checkup may result in an inconvenience and a health threat for them [6].

Ambient Assisted Living (AAL) [7] has emerged as an appealing alternative to face this circumstance by taking advantage of the latest advances in research and technology such as the Internet of Things (IoT) paradigm, Wireless Sensor Networks (WSNs) and low-power consumption hardware platforms, to name a few. Hence, patients can be provided with an AAL environment aimed to detect domestic accidents (e.g., falls, flooding, fire, etc.), monitor their health, help them on their daily tasks, and, overall, improve their quality of life [6].

This is especially targeted to those patients who have a reasonable degree of both physical and mental autonomy to carry on regular Activities of Daily Living (ADL) [6], but, because of their age or disease, need to be continuously monitored (e.g., see [8,9]). In this regard, AAL-based solutions typically aim to radically transform (or even build from scratch) the home of the patients by installing a bunch of sensors, robots or wearable devices [6]. However, they often result in prohibitively expensive ad-hoc systems that are hard to deploy and maintain at large scale [10]. Additionally, these solutions present several concerns that make people apprehensive of using them [10], such as: privacy (e.g., cameras recording the patients), usability, technological complexity and expenditure, etc. Hence, cost and intrusiveness play a crucial role when designing and deploying an AAL-based system aimed to be adopted by a broad range of people.

**Figure 1:**
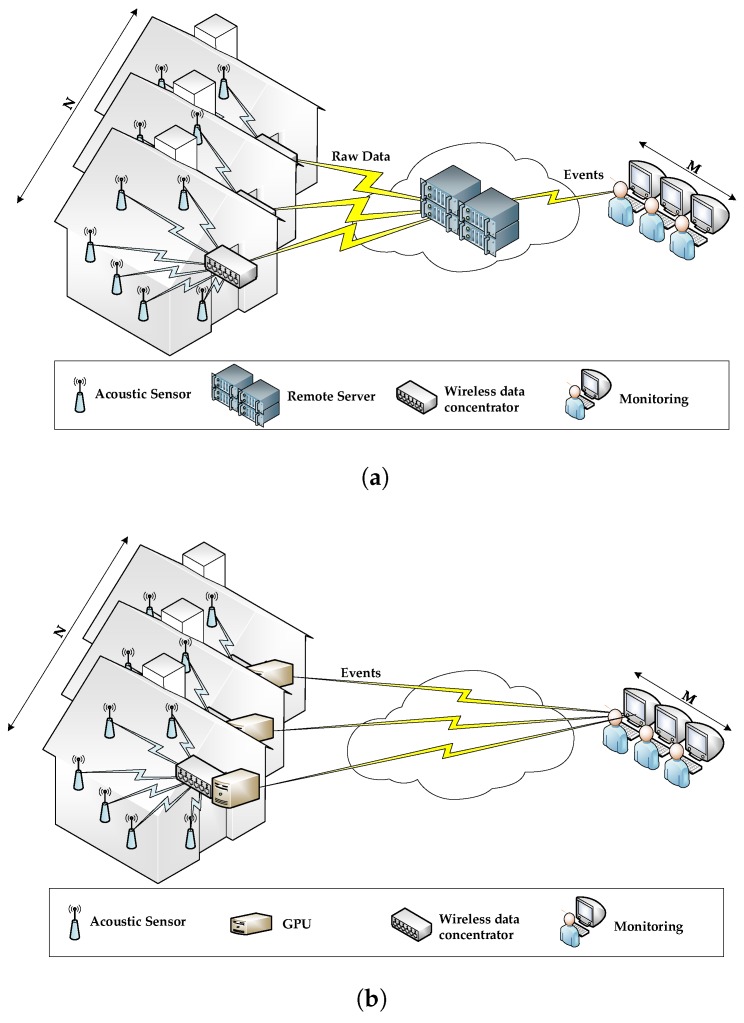
Architecture alternatives for a tele-care system to remotely monitor an acoustic-based Ambient Assisted Living (AAL) environment. (**a**) Centralized intelligence architecture; (**b**) Distributed intelligence architecture.

Acoustic interfaces have become a convenient solution to enable older patients to interact with complex AAL environments, since they are perceived as non-intrusive [11], and can be easily integrated to the existing living environment. Specifically, they can serve to multiple purposes, like acoustic activity and audio event detection, sound source localization or speech recognition [12], to name a few. When considering where to deploy the computing power of a remotely monitored AAL environment, there are two major alternatives as shown in Figure 1: *(a) centralized intelligence* where the nodes are used for sensing and transmitting the raw information to a central computing host (or remote server) that will be able to process such amount of data nodes, as in Figure 1a, and *(b) distributed intelligence* where the nodes of the network have their own computing capabilities that enable them to implement the required algorithms locally, as in Figure 1b.

The centralized strategy (Figure 1a) is based on deploying a Wireless Acoustic Sensor Network (WASN), where every node with a microphone sends all the acquired raw data to an intermediate remote server that is asked to process them according to the target application (e.g., monitoring, surveillance, event recognition, etc.). However, these remote servers can become a bottleneck due to the throughput and volume of data to be collected and processed (i.e., *N* homes × *K* sensors/home × 24 h/day × 365 days/year). Also, considering the real-time and reliability constraints imposed by this kind of applications (i.e., both the servers and the communication links need to be operating 100% of the time), the degree of redundancy and the communication protocols needed to meet these requirements would result in an expensive solution [13]. Additionally, in this approach very sensible personal data are stored in the cloud, which makes them prone to cyber attacks and, thus, would require an expensive additional layer of security [14,15].

Alternatively, Figure 1b shows a distributed architecture inspired by the Mobile Edge Computing (MEC) paradigm [16] where low-cost computing platforms (e.g., Arduino, Raspberry, programmable logic devices, Graphics Processing Unit (GPU), etc.) are deployed at each indoor environment to process the raw data acquired by the WASN. Then, each device only sends to the monitoring system the encrypted acoustic event identifier. This approach addresses both the burdensome communication costs and the principle of privacy [15] simultaneously in a cost effective way. Overall, this strategy can also be seen as a MEC architecture where the cloud computing infrastructure consists of a single computing node and user equipments (i.e., microphones) have no computing capabilities [17].

The purpose of this paper is to take advantage of this latter approach to design acoustic-based AAL environments [18,19] aimed to enhance the life quality of the elderly by addressing the key requirements of both users and care providers: being non-intrusive, allowing to keep personal data privacy, being easily deployed at home, permitting robust remote monitoring in real-time, and last but not least, being affordable (both in terms of communications and hardware costs).

As a proof-of-concept, the proposal is implemented using a high performance General Purpose Graphics Processing Unit (GP-GPU) and considering an audio database with 14 different sound typologies derived from daily life activities. This way, we study the viability of remotely monitoring—via a tele-care system—the patients behaviour by tracking certain sounds at home (e.g., water-related events, doors closing, etc.), besides detecting emergency situations via acoustic event recognition (e.g., falls, glass breaking, etc.) in real-time, with a reasonable cost and accuracy.

Although a specific medical application has not been designed yet, we build on previous investigations where acoustic event detection and behavioural tracking following water noise events were used to detect early stages of dementia [8,9]. In this work, we combine this kind of acoustic data with other audio events derived from ADL at home, which can help doctors to identify rare behavioural patterns that with a regular hospital visit are unnoticeable to the professionals (e.g., someone frying food at unseemly hours), besides allowing the detection of hazardous situations (e.g., a baby crying, a glass breaking) in real-time.

Hence, the main contributions of this work are the following:

1. A detailed state-of-the-art review of existing AAL techniques based on audio processing and high performance computing platforms.
2. The design and deployment of a low-cost distributed architecture at home to collect and process audio data from several sources based on a low-cost GPU embedded platform.
3. The implementation of a data-mining based system able to detect and classify acoustic events related to AAL in real-time.

The remainder of this paper is organized as follows. Section 2 reviews the related work on AAL and audio events characterization. Section 3 details the proposed architecture to collect and process audio data in in-home environments, and Section 4 details the event detection algorithm and its stages. Section 5 presents the conducted experiments. Section 6 discusses the results of the experiments in relation with the related work. Finally, Section 7 concludes the paper and draws some future work directions.

## 2. Related Work

In recent years, there has been a rapid evolution of AAL related technologies due to the gradual aging of society, aiming to provide care and support to older adults in their daily life environment. These technologies include smart homes, assistive robotics, wearables and smart fabrics, to name a few. Several projects have already dealt with the challenge of collecting and processing real-life data at home, and defining personalized actuators adapted to the particular needs of this segment of the population. This section reviews the state-of-the-art of AAL applications, including some representative platforms used to process the data at home, giving special attention to those WASN designed for this purpose.

### 2.1. AAL Research Projects

A smart home can be defined as a regular house with several sensors installed to measure various types of data [20]. Ambient information can be obtained by analyzing the data collected by the sensors [6,21]. Typical interesting collectible home data includes motion, identification, pressure measurement, opening/closing of doors, kitchen activity, etc. The collection of data has been usually performed by infrared motion sensors, RFID, pressure sensors, smart tiles, cameras, ultrasound sensors, microphones and magnetic switches. The reader is referred to [22] for a recent survey presenting a complete insight to the different types of sensors in the context of AAL. Several projects have been conducted within the AAL framework, and we have classified them to be named in function of their final goal. We first enumerate a group of projects focused on allowing people to age at home, being some of them funded by the Assisted Living Joint Programme [23]. The project Aware Home [24] uses a wide variety of sensors, covering from specifically designed smart floors to more typical video and ultrasonic sensors, together with social robots to monitor and help older adults [25]. The Ubiquitous Home Project [26] is also centered in residents monitoring through a robot, which includes a dialog-based interface and operates as an intermediate platform between the smart home and the end users. This is one of the AAL projects that involves the deployment of microphones at home, to allow voice-based interaction between the users and the robot.

Other projects have been focused on the monitoring of people suffering from a certain disease. The CASAS project [27] uses a smart home to monitor people suffering from dementia, which is also the goal of the previous ENABLE project [28], which installed and supported an internationally distributed equipment to monitor people with dementia, with the goal of giving them more autonomy in their lives. Finally, the DOMUS [29] and IMMED [30] projects pretend to recognize the behavior of a person suffering from Alzheimer’s disease in an early or intermediate stages.

Another topic of interest of AAL projects during the last years has been behaviour or activity monitoring. The Elite care project [31] focuses on the fact that changes in motor activities patterns during sleep can be a disease indicator, or at least, they can reflect abnormal physiological and neurological conditions. Project House_n [32] presents an alternative to track the house activity using sensors like cameras or microphones, which need a signal processing computation to derive behaviour conclusions. Grenoble Health Smart Home [33] supplies a set of tools for the measurement of patients activity in hospitals via several indicators of their activities in daily living, mobility, agitation, repartition of stays and displacements. In the Gloucester Smart House project [34], a tele-care system was designed, based on lifestyle monitoring, with the pretension of continuously gathering information about the person’s activity during daily routines. Some of these systems also sample biomedical constants, like in the Welfare Techno House project [35], which collects automated electrocardiogram measurements in bed or in the bathtub, without any awareness of the subject. In the Aging in Place project [36], residents are tested for urinary tract infections, and alerts are sent to the care coordinator using a sensor network; the goal is to detect signs of illness earlier than traditional health care assessment.

### 2.2. WASNs for Tele-Care

A WASN is a group of wireless microphone nodes spatially distributed over an indoor or outdoor environment. Its design has to take into account the scalability of the network, the delay of the acoustic signal, the synchronization of the nodes and the decision of where the computing is performed [12] (see Figure 1). One of the applications of sound source localization is the positioning of the person living alone [37] by means of a central system that aligns and analyzes the data coming from all the sensors (see [38] for more details). Another typical application of WASNs deployed in AAL environments is Acoustic Activity Detection (AAD) [12]. The primary purpose of AAD is to discriminate the overall acoustic events from the background noise [39], overcoming those approaches only based on energy threshold detector. Among the AAD, Voice Activity Detection plays a significant role for AAL solutions including acoustic interfaces [12].

Moreover, several works that include acoustic signal processing have undertaken a multimodal approach. In [40], the authors present a preliminary study focused on the recognition of clinically relevant activities in the daily life of elderly people using a low-power WASN, composed of audio and ultrasound sensors. In [8], the goal of the proposal was to detect early stages of dementia in elderly living at home, using the audio and video samples recorded in the house. In [41] up to 21 different sounds occurred in the kitchen are distinguished to define the patients behaviour. More recently, [42] has considered sequence-based models for performing automatic human activity recognition in voice-controlled smart homes equipped with non-visual sensors (e.g., such as acoustic sensors), actuators and automation equipment.

Acoustic sensors at home can also be used for surveillance applications when taking care of the elderly or the disabled [43]. In [44], an acoustic fall detection system oriented to the elderly age group living at home is described. Doukas and Maglogiannis also presented in [45] an emergency falling detection system. The latter approach utilizes video, audio, and motion data captured from the patients by means of body sensors and their surrounding by using overhead cameras and microphone arrays; their proposal also analyzes post-fall data so as to determine the severity of the fall. Finally, the CIRDO project [46] was another multimodal effort to build a healthcare system to ensure the safety of seniors and people with decreasing independence at home. To that effect, CIRDO implements an audiovisual system that runs standard audio processing and video analysis tasks on a GPU. Despite the project’s effort of privacy and private data, the patients are still not comfortable living with a system that processes real-time the video of the home activity.

### 2.3. Embedded Audio Applications Using Low-Cost Graphical Processing Units

Beyond the aforementioned projects, several works have also addressed the need of intensive and real-time processing of audio signals for AAL purposes. High-end GPUs, with thousands of cores per-chip, have been used in High Performance Computing (HPC) applications due to their excellent performance in parallel computing of multisensor systems, in comparison with other low-cost high performance platforms (the reader is referred to [12] for further details). The main drawback of these platforms is that their power consumption and cost do not fit some of the critical requirements of most embedded system applications, such as [47]: (i) power consumption; (ii) mobility; (iii) size; and (iv) cost. Taking into account the satisfaction of these restrictions, in this work we propose to use the NVIDIA Jetson TK1 (Tegra K1) [48], designed with an Advanced RISC Machine (ARM) cortex-A15 and a NVIDIA Keppler architecture GPU with 192 cores, which allow to use this HPC devices in an embedded system. This low-cost platform has shown benefits in topics such as computer vision, robotics, automotive, image signal processing, network security, medicine and many others [49].

An application using spatial audio rendering based on Wave Field Synthesis (WFS) was presented in [50] to emulate virtual sounds, including a minimal multi-processor architecture using a Field Programmable Gate Array (FPGA). Their experimental results suggest that the Virtex4FX60-based FPGA prototype, running at 100 MHz, could provide a kernel speedup of up to 4.5 times compared to an OpenMP-annotated software solution implemented on a Core 2 Duo at 3.0 GHz. Later, this study was extended using a GPU [51], which led to the conclusion of the suitability of this device for spatial audio rendering. Based on their conducted experiments, both high-end GPUs and FPGA solutions are good choices to develop complex Immersive-Audio systems utilizing tens of input and output channels. Besides high performance, both can provide more power-effective solutions compared to traditional PC-based designs. In [52], WFS is also applied over a GPU but with a higher number of sound and speakers’ sources (around 200), taking advantage of the GPU parallelization capabilities (in this case, a NVIDIA C2075 GPU). Finally, an improved version of the same approach is detailed in [53], which outperforms previous results thanks to the inclusion of room compensation techniques. This system is implemented with a Kepler GK110 GPU, which has the same architecture as Tegra K1 systems-on-chip (SoC) that is embedded in the Jetson TK1 [48] development kit.

A real-time audio-to-score alignment based on dynamic time warping (DTW) has been implemented recently in [54] for multi-core architectures (x86, x64 and ARM) and MALI ARM and TK1 NVIDIA GPUs.

In [55] an automatic environmental sound recognition system is described using the Mel frequency-cepstral coefficients as audio features considering three different classifiers: (i) Gaussian Mixture Models (GMMs); (ii) Support Vector Machines (SVMs) and (iii) Deep Neural Networks (DNNs). The comparison of these classifiers is done in terms of computational power and classification accuracy after being embedded in a NVIDIA Tesla K40c GPU. The authors conclude that DNN outperform both GMM and SVM in terms of performance, but in terms of computational load, the number of hidden units and the number of layers has to be limited to avoid computational load to be much higher than the other two approaches.

Finally, it is worth noting that the emergence of GPU computing on embedded systems provides a good opportunity to develop computer vision applications with low cost. In [56], a tele-presence application based on face detection is presented using the TK1 GPU that is optimised to provide an speed up of x10 compared to an ARM Central Processing Unit (CPU). A GPU-based face detector using the Non-Maximum Suppression algorithm is described in [57], showing a latency reduction compared to other CPUs. Ulusel et al. [58] present a power and performance comparison of three feature extraction techniques used in computer vision application; after evaluating their performance on different embedded systems (embedded CPUs, GPUs and FPGAs). The authors conclude that FPGAs outperform GPUs (both outperforming CPU) for the task at hand because of the memory access bottlenecks and the high number of kernels required for this kind of implementation. These results could be swept if the data to be processed turned from being images to plane signals (e.g., presence, acoustic, etc.), due to the total amount of data that is transferred to the GPU to be processed [59].

## 3. WASN for Surveillance and Behavioural Monitoring

The architecture of the tele-care system is designed to be capable of gathering and processing the acoustic information in real-time to allow remote patients’ surveillance monitoring (e.g., to attend emergency situations as soon as possible) and behavioural tracking (e.g., to detect specific diseases in early stages). To that effect, the system follows the architecture described in Figure 1b fulfils the principles of non-intrusiveness and privacy in a cost effective way. Furthermore, embedded systems allow the development of applications focusing on different aspects such as (a) dependability; (b) efficiency and (c) real-time constraints, which will be met in this proposal.

### 3.1. System Architecture

This MEC-inspired proposal [17] consists of the deployment of a WASN at home or in residences able to gather the data of all acoustic sensing nodes deployed to infer the audio events of interest. It follows a distributed implementation, where all digital signal processing is carried out in a concentrator offloading the sensor nodes and avoiding the use of the server to remotely process the data [16,17]; the remote station only monitors the acoustic events recognized and transmitted by this concentrator (see Figure 1b). In the audio event detection module, three key elements can be distinguished: (a) several wireless acoustic sensors; (b) a concentrator with the GPU and (c) remote monitoring.

**Figure 2:**
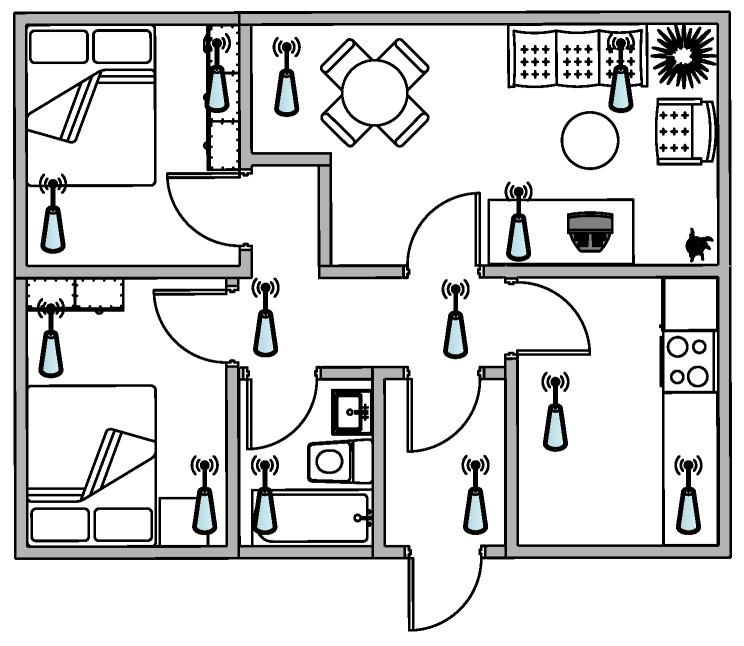
Example of wireless sensor deployment at home or in a medical facility.

The system relies on a wireless network of sensors distributed around the house (see Figure 2). The microphones have to be installed in such a way that they present small correlation between them to obtain space diversity. Each sensor is based on a microphone to record the environmental sound and wireless connectivity to transmit the information taken by the sensor to the concentrator, where the raw information will be processed. The used microphones present a good trade-off between the frequency response and cost; tests have been conducted with the elected condenser low-cost microphone CMA-4544PF-W [60] of the manufacturer CUI Inc (20050 SW 112th Avenue Tualatin, OR 97062). Notice that some works have also considered using acoustic sensor arrays, however, they have not been considered in this work since they sample a sound field locally [12]. In this way, each microphone transmits sounds to this device that acts as a concentrator—the core element of our proposal. As a matter of fact, this concentrator (i) collects all the audio sounds of the house; (ii) processes them in order to extract their features by means of a GPU; (iii) infers the source of the audio event; (iv) performs the network signal processing of all the sensors; and finally, (v) sends this information to a remote server that monitors the needs of the people living in the house. The concentrator needs to take advantage of parallel capabilities due to the high number of sensors to be processed individually and in the network; for this reason a deep analysis of the different algorithms needed in this work has been done in a co-processing GPU [48,49] in order to speed up the execution time.

### 3.2. GPU for Acoustic Signal Processing

Microprocessors based on a single CPU have improved their performance during two decades by means of increasing the clock frequency. However, this increase has slowed down since 2003 due to heat dissipation and power consumption [61]. Currently, microprocessor architecture designers have mainly focused on both (a) the multicore approach, multiplying approximately by two the number of cores in every generation maintaining the clock frequency; and (b) the many-core approach, which has focused on the execution throughput of parallel applications through the use of a very large number of smaller cores [62].

**Figure 3:**
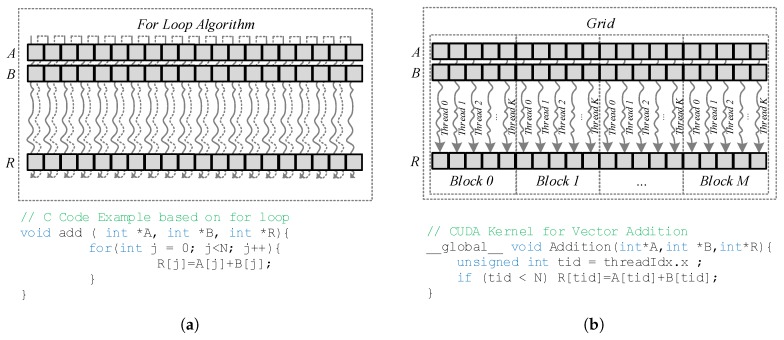
(**a**) CPU operation performance, (**b**) GPU operation performance.

The main difference between a CPU and a GPU lies in their architectures. CPUs have been optimized to carry out sequential code efficiently while GPUs are able to launch thousands of threads in parallel, depending on the platform and the resources requirements, executing the *same piece of code* with different data. For instance, a single-core CPU that adds vectors will do that step-by-step conducting a single operation in every iteration of the loop, as we can see in Figure 3a, while a GPU may be able to do that at once, in a single step, launching the same number of threads that the length of the array where every one performs the operation of a single point of the memory (see Figure 3b). For this reason, the execution model of the NVIDIA GPU is called Single Instruction Multiple Thread (SIMT) [63].

NVIDA Jetson TK1 [48] is an embedded development kit based on the NVIDIA Tegra K1 System on Chip (SoC) which is composed of (*a*) the NVIDIA Kepler GPU with a single streaming microprocessor (SMx) with 192 cores and (*b*) a quad core ARM cortex-A15 CPU. Tegra family is the proposal of the NVIDIA manufacturer for mobile processors in which you need GPU-accelerated performance with low power consumption. Kepler architecture offers an improvement of performance up to three times more than previous generations, Fermi [64]. This level of concurrency allows us to process audio events of several sources in real-time. NVIDIA supplies this development kit with the full Board Support Package (BSP) which comprises Compute Unified Device Architecture (CUDA), OpenGL 4.4 and OpenCV API.

## 4. Audio Event Detection Algorithm

**Figure 4:**
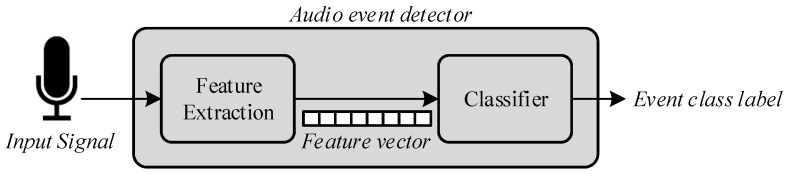
Event Detection Algorithm Steps.

The audio event detection algorithm consists of two different stages, as detailed in Figure 4: feature extraction and classification. Feature extraction is a signal processing procedure that aims to parametrize the key characteristics of the audio events by means of a set of representative coefficients with a lower dimensionality than the original samples [65]. Once these coefficients are available, they are input to the analysis and classification module, to obtain the identification of what type of acoustic event has occurred of the predefined universe of interest. The two phases of the event detection process are further detailed below.

### 4.1. Feature Extraction

In order to characterize the audio samples, we have used the well-known Mel Frequency Cepstral Coefficients (MFCC) [66] based on the perceptual Mel scale [67], following previous approximations [18,19] due to their promising results. In [18] first tests were conducted using MFCC, and in [19] the tests with MFCC were implemented over a GPU.

**Figure 5:**
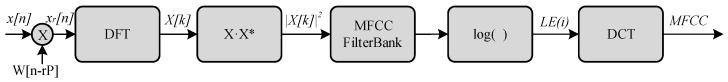
Mel Frequency Cepstral Coefficients extraction from the raw acoustic signal.

The method to compute MFCC is shown in Figure 5. The incoming audio signal is divided into blocs of 30 ms with a sliding Hamming window to improve the frequency resolution of the Discrete Fourier Transform (DFT). The sliding window has an overlap of 50% of samples to compensate the signal reduction due to the Hamming window. These frames are transformed into the frequency domain using the DFT to evaluate the contribution of every band of the spectrum. This step uses a filter-bank of 48 Mel scale filters. The final coefficients are obtained after extracting the first 13 coefficients of the Discrete Cosine Transform (DCT). The high order coefficients are discarded to reduce the parametrization dimensionality due to the fact that the main information of the acoustic event can be found in the lower DCT region, which corresponds to the signal envelope [66]. As a result, each 30 ms audio signal frame is characterized with a vector of 13 components, which is fed to the classifier.

### 4.2. Automatic Audio Event Classification

The audio event classification module aims at automatically determine to which event corresponds a certain 30 ms audio sample. It works with the 13-component vectors resulting of the MFCC feature extraction module and outputs the inferred audio event label that this vector comes from, using an automatic classifier based on semi-supervised learning.

Automatic audio classification has been studied for decades [68] and several methods have been proposed so far (see [69] and references therein), ranging from decision trees to unsupervised learning strategies. However, acoustic event classification has some particularities that prevent us from using existing general approaches. Specifically, we have bumped into the following concerns:

- Class overlapping [70]. Splitting acoustic events in quasi-stationary short frames enriches the characterization of events but drives to a situation where two 30 ms samples from different events might share several similarities in terms of their spectrum and, hence, the derived MFCC values.
- Samples coherence. The same audio event can be defined by different combinations of a fixed set of 30 ms frames. That is, a glass breaking can be characterized by the sequence of 30 ms frames *A*-*B*-*C*-*D*, *A*-*C*-*B*-*D*, etc. Unfortunately, artificially building a data set with all possible combinations as suggested in [69], is not feasible due to the huge amount of samples to combine. This is very different from other audio domains such as speech recognition where the system has to identify fixed audio sequences, which follow a phonetically-based temporal evolution [65]. Therefore, existing approaches based on Hidden Markov Models (HMM) [69] do not fit well the problem at hand [71].
- Class imbalance [70,72]. The length (i.e., number of vectors) of each audio event is different (e.g., someone falling lasts longer than a dog barking). Existing solutions that aim to sub-sample or over-sample the original data cannot be applied to the audio domain because the semantics variation of the derived MFCC would affect the classification performance negatively.

**Figure 6:**
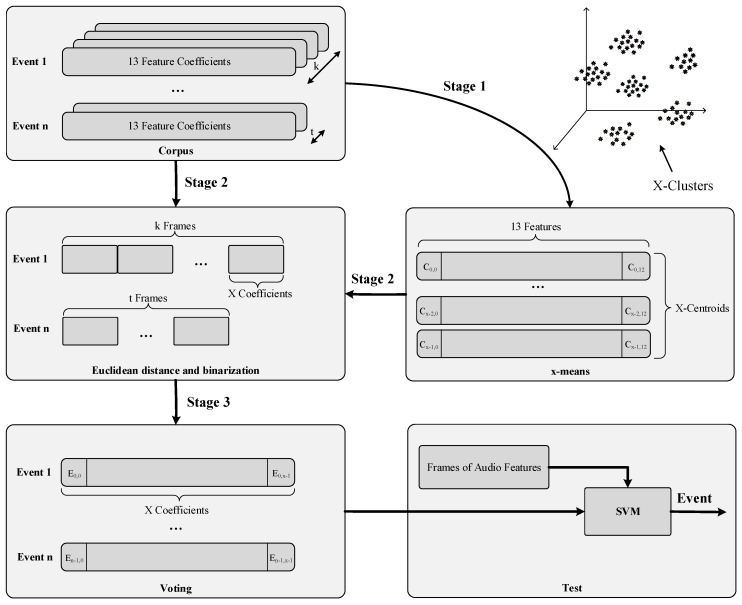
Training process of the classifier.

To address these issues, we have split the training process of the classifier in three stages as shown in Figure 6. At the first stage, the centroids of the subspace defined by all the 13-components feature vectors collected from the labelled audio database are obtained. The optimal number of centroids (*X*) is evaluated with the x-means algorithm [73], while the positions of the centroids are obtained from the k-means algorithm [73]. Note that putting all the centroids together results in a space transformation matrix ($M_{st}$) of *X* rows by 13 columns—*X* for the new space dimensions and 13 for the base subspace dimension. At this point, the class overlapping issue has been addressed since the samples are transformed to a new space with another dimensionality (*N*) that emphasizes their slight differences.

At the second stage, the audio events are transformed to the new space using the aforesaid $M_{st}$ matrix. Specifically, each of the original 13-components vectors of an acoustic event are compared to each of the dimensions of the new subspace to find out which is the more similar one. The vector that characterizes the acoustic event is built upon counting how many 13-components vectors are similar to each dimension of the new subspace. As a result, a normalized *N*-components vector for each acoustic event—disregards its temporal length—is obtained. This vector models the acoustic event in the new subspace. At this point, the samples coherence issue has been addressed thanks due to the fact that the building process of the new vectors only takes into account the number of occurrences instead of their temporal sequence. Additionally, the class imbalance issue is also addressed since all the events have the same length (i.e., *N*).

Finally, at the third stage the obtained vectors are used to train a Support Vector Machine (SVM). This stage is aimed at generalizing the knowledge represented by these input vectors. Given the potentially high dimension of this data set and the high number of classes, we have selected a linear SVM [74] and a one-against-one [75] coding scheme to cope with this multiclass problem. Basically, we have built E(E−1)/2 SVMs (being *E* the number of events to be detected) where each SVM classifier is trained with data from two classes, which limits the class imbalance problem typically found using the one-against-all method [75]. To find out the best values for the configuration parameters of each SVM (i.e., the *C* constant) we have held-out a 10% of the training set and conducted an exhaustive grid search with cross-validation. Note that prior training any SVM, we have conducted a soft normalization of the training set to prevent attributed with great numeric range from dominating over the smaller numeric range ones. This soft normalization consists of the following: for each attribute we have subtracted the means of its dimension and divided it by twice the standard deviation (adding a small epsilon to prevent divide-by-zero errors). At the test stage, the input vectors will be also normalized in the same way (i.e., we keep the means and the standard deviation of the training set).

When the system is set in exploitation mode, the vectors coming from the feature extraction module are transformed to the new subspace using the process depicted at the second stage, normalized, and submitted to the SVM system to obtain the predicted class (i.e., audio event). At this test stage, a voting between all SVMs is conducted to find out which is the majority class: if $sign({\left(w^{ij}\right)}^{T}\phi \left(x\right)+b^{ij})$ is positive a vote from *x* is added to the *i*th class, else a vote is added to the *j*th class [75].

## 5. Test and Results

In order to assess the feasibility of our approach, we have conducted a proof-of-concept experiment, which is analyzed in terms of algorithm operations and performance. This experiment is aimed at detecting 14 common in-house audio events derived from ADL with a NVIDIA Jetson TK1 (Tegra K1) [49] for patients’ surveillance monitoring and behavioural tracking. In this regard, we have selected the following representative 14 audio events: someone falling down, a knife slicing, someone screaming, rain drops, a printer working, people talking, someone frying food, someone filling water in a pot, someone knocking the door, a dog barking, a car horn, a glass breaking, a baby crying, and water boiling. Note that this event selection covers several interesting cases from the AAL and audio processing domains, which allows us to test the performance of the proposed classification algorithm on a diversity of situations (e.g., alarm events such as someone screaming, dog barking; tracking events such as frying food, filling water; impulsive sounds like dog barking, door knocking, glass breaking; or pseudo-stationary sounds like water boiling, baby crying, printer working). Therefore, we fill confident that the collected results in this experiment can be valuable for the implementation of a surveillance and behaviour monitoring application in the real-world. To conduct the tests, we have collected a total of 154 audio samples (11 corresponding to each event) from the freesound (freesound.org) database resulting in 23.521 frames of 30 ms. Note that the number of frames corresponding to each audio event will vary according to the length of the acoustic sample.

### 5.1. Analysis of the Algorithm Operations on the GP-GPU

The proposed audio event detection algorithm entails a considerable computation cost overhead, which envisages the usage of a high-performance computing platform. In this section, the computational cost in terms of floating points operations (FLOPs) of the whole algorithm when set in exploitation mode (i.e., after being trained) is analyzed.

As detailed in Table 1, we have considered that for every audio frame: (i) *M* is the number of samples from the Analog to Digital Converter (ADC) within a 30 ms frame; (ii) *N* is the next two’s power and (iii) *R* is the number of the filter banks; (iv) *D* is the number of Mel coefficients obtained, feeding the SVM; and (v) *E* is the number of events to be detected. The proposed implementation detects 14 events (E=14), samples the audio source at 48 kHz (which corresponds to M=1440 samples and N=2048) and uses 48 filter banks (R=48), obtaining a total of D=13 Mel coefficients.

As shown in Table 1, the most expensive operations are the filter bank, the Fast Fourier Transform (FFT)—as the implementation of the DFT—, and the SVM (only in those situations with hundreds of events to detect). Note that, the operations associated to these algorithms can be easily executed in parallel by running them in different threads. As the real-time constraint is a mandatory requirement for the tele-care application at hand, the usage of a GP-GPU enables us to (i) carry so many operations in a cost-effective way and (ii) process several audio sources in the same house in terms of acoustic network signal processing.

It can be shown that the order of magnitude of the number of required FLOPS to conduct the feature extraction and classification is below the maximum afforded by mid-range GP-GPUs as the NVIDIA Tegra TK1, which shall enables us to include more audio sources and process them in parallel. In this regard, an appropriate execution pipeline and the cache usage should be carefully analyzed, which is out of the scope of this paper.

### 5.2. Analysis of the Algorithm Performance in terms of Audio Event Detection Accuracy

Definitely, the high number of parallel operations that can be parallelized (e.g., Filtering, FFT, matrix multiplications) in the proposed approach makes it very suitable to be executed in a GP-GPU. Therefore, for the sake of this proof-of-concept, the proposed audio event classification system has been deployed on the Tegra K1.

To test the overall accuracy of the proposal and make the experiments significant for the surveillance and behaviour monitoring applications, we have used the aforementioned 14 events as the input data set. That is, we have copied the audio files to the board and run the classification algorithm as detailed in what follows.

**Figure 7:**
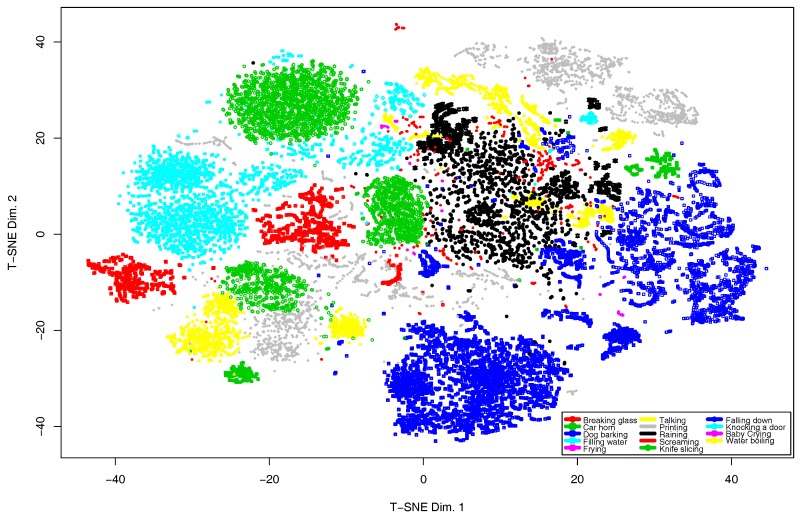
*t*-SNE plot of the training data set.

First, to figure out the distribution of the training data set, we have conducted the *t*-Distributed Stochastic Neighbor Embedding (*t*-SNE) [76] analysis, which results are depicted in Figure 7. From this plot, it can be observed that there is an event overlapping in the following classes: raining and water boiling, filling water and knife slicing, printing and car horn.

To address this concern, we have improved the classifier described in Section 4.2 following a hierarchical approach. The process follows the next steps: (i) it compacts each pair of overlapping classes into a single class; (ii) it reorganizes the audio database with the compacted classes; (iii) it trains the classifier with this database; and (iv) it trains an individual classifier for each pair of overlapping classes.

Next, we have trained the classifier using the 60% of the samples and tested the system accuracy with the remainder 40%. That is, we have randomly taken the 60% of samples for each class to train the learning classifier system and test its accuracy with the remainder 40% of samples for each class. After testing the system with 10.000 runs and conducting a 10-fold cross-validation, we have obtained the results shown in the confusion matrix at Table 2. Each row of Table 2 is associated to an acoustic event. Columns represent the competence of the classifier when classifying this event in front of the others. That shows how many times an event (i.e., rows) has been confused with other events (i.e., columns).

We can see that the overall accuracy of the system is close to the 82%. Also, it can be observed that the audio events with a short temporal length (e.g., falling down, knife slicing) obtain the worst accuracy due to the fact that there is not enough entropy on the samples in the test database to characterize them. It seems reasonable that building another hierarchical classifier after compacting these classes (e.g., falling down, raining and knocking a door) would improve these results.

Overall, these results show the feasibility of our approach and encourages practitioners to work in this direction with richer data sets (in both the number of samples and the number of audio events) and more sophisticated classifiers that may outperform the linear SVM herein used, with an affordable computational cost for platforms such as the one used in this work.

## 6. Discussion

So far, we have obtained what to the best of our knowledge are impressive results when processing audio data collected from a WASN with a GPU. After demonstrating the feasibility of our proposal for tele-care applications in the proof-of-concept described in Section 5, we would like to share some lessons and experiences collected during the design and development stages of the platform that might contribute to improving future versions of this platform.

### 6.1. Robustness of the Real-Time Audio Events Classification

Reliability and robustness of the audio event estimation are two of the key aspects to be addressed when designing any acoustic monitoring platform. For this purpose, an exhaustive study of several feature extraction techniques should be conducted taking the MFCC results as baseline, in order to determine which type of parameters are more suitable to represent the audio events to be discriminated. Moreover, different data mining algorithms should be compared thoroughly as classification is a key point for this kind of machine hearing systems. Finally, we have to assure that the data mining algorithm is trained using an audio database that ensures enough diversity for each category of sound events to be identified.

Another critical issue related to audio event detection is facing real-life implementation changes. Even when the system is properly trained with real data recorded in the same place of operation, running in a real-world environment means that some situations beyond the ones observed in previous experiments might happen [77,78] (e.g., the occurrence of sound events out of the universe used to train the classifier that may led to lower accuracies, the presence of a larger diversity of Signal to Noise Ratio (SNR) in the raw acoustic signal recorded by the sensors during the training stage). Therefore, some sort of threshold should be included to the classification process (e.g., limiting the maximum distance between a sample and the closest centroid) in order to avoid false positives.

#### 6.1.1. Event Overlapping in Groups of Classes

As detailed in Section 5, it is essential to conduct a preliminary study of the universe of audio events to be detected in order to observe potential overlaps between them. In this work, we have detected three pairs of events with higher overlapping than the others, which has led us to modify the initial classifier to separate these classes and use a hierarchical approach.

As a general methodology, every time new audio events derived from patients’ activities are added to the database, either to enrich the existing samples or increase the number of sounds to be identified for patients’ remote monitoring, their overlapping with the previously recorded events has to be analyzed, and the classifier has to be adapted accordingly to keep a good performance in the new universe of data.

#### 6.1.2. Audio Events Mixture

This proof-of-concept presents results from tests performed over an audio database where each audio sample corresponds to a single ADL acoustic event. In real life operation, audio event overlapping should be also taken into account (e.g., dog barking while boiling water). For an optimum performance of the system in its two tele-care applications (i.e., for surveillance and for patient behavior monitoring), it is recommendable to use a classifier able to identify more than one coexisting event at a time. In this regard, data mining algorithms providing more than one output [79] that rely on the audio event probability of occurrence should be considered. In fact, the mixture of audio sources and its separability is one of the hot research topics within the acoustic event recognition community [80].

### 6.2. Acoustic Nodes Placement

Audio signal processing offers several advantages in terms of indoors coverage because it does not require visual or physical interaction with the sensor to record the data of interest. Despite that, in an indoor environment, loud noises propagate by diffraction when there is no line of sight, thus, it is fundamental to carry out a complete study of the location of each of the acoustic nodes and their coverage in their position. The WASN has to be deployed carefully enough to ensure that there are no blind acoustic spots in the place to be monitored [12]. It is also recommendable to consider slight redundancies in the acquisition to increase the robustness of the audio event detection. That is, if more than one node detected the same event, the error probability in the classification should decrease. Also, if the same audio event is detected by several sensors with different signal to noise ratios, such information can be used to coarsely detect its location [12].

### 6.3. GP-GPU Platform Proposal

Taking into account the computing capabilities of existing GP-GPUs exhibited in the proof-of-concept, it might seem that these devices are over dimensioned for audio data processing coming from a single source. However, we reaffirm on our proposal to use the NVIDIA Jetson TK1 for the described tele-care application because in a real network environment, it should deal with a larger number of acoustic sensors—as many as needed for the correct coverage of a home or a residence—and this necessarily leads to an increase of the computational load of the acoustic signal processing and classification stages.

This comes from the fact that the acoustic signal processing and its classification are computed locally using the GPU of the NVIDIA Jetson TK1. In order to provide all this information in real-time (i.e., to obtain results within the recording time of the following frame), it is necessary for the computing device to support a massive processing of audio data. In fact, for both surveillance and monitoring applications, the information must be properly timed and notified in the occurrence moment to take the appropriate actions in case of emergency. This drives us rely on an embedded platform based on a GP-GPU that offers high performance computation features.

To sum up, every acoustic node raw signal in the indoor environment has to be carefully processed: from considering possible audio mixtures to selecting the best audio coefficients (i.e., features) that characterize it. Additionally, to increase the system robustness and improve its accuracy, the identification of an acoustic event can be supported—or unsupported—by other redundant sensors deployed over the environment by using a voting weighted voting strategy (e.g., weights of closer sensors are higher than others). Then, the result of the voting is sent to the tele-care system, which receives a conclusion of the situation at home, both in terms of surveillance and in terms of behavioural monitoring of the person. From this information, the tele-care system will activate the appropriate actions if necessary.

### 6.4. Implications of the Proof-of-Concept

The proposal of tele-care system presented in this work should include in future implementations a decision-making process or a decision-support system, depending on the activities of daily living that should be detected. This is especially important when a detected event implies the activation of an alarm (in a surveillance situation at home), but also when the goal is to remotely monitor the behaviour of people so as to conclude whether there is an abnormal situation at home or not.

In this regard, it would be very useful to incorporate a long-term analysis within the audio event detection algorithm beyond the current short-term analysis. As a result, the latter classifications would give information to the tele-care system of what it is happening at the moment and also about the urgency of support requested if necessary, while the former would allow to analyze and track the behaviour of the patient over time.

Finally, it is worth mentioning that the described system can be fully personalized according to the care plan and user profile, covering from elderly aging at home mostly autonomous, to adults with a certain degree of dependence who prefer to live in their own space but need some kind of support.

## 7. Conclusions and Future Work

This paper presents a proof-of-concept of a tele-care application proposal using a NVIDIA GPU of a Jetson TK1 board to collect and process the acoustic data from a WASN deployed in a home or a residence. The aim of this work is to address two important concerns in AAL: behavior and surveillance monitoring. On the one hand, it allows detecting emergency situations (e.g., somebody falling, a glass breaking), and on the other hand, it enables to monitor patients’ behaviour by tracking the occurrences of certain sounds. The GPU high computing performance allows (i) preservation of the privacy of the collected data from users by means of locally processing all audio signals in parallel and (ii) informing the care provider about the patient status in real-time.

Conducted experiments in this proof-of-concept demonstrate the feasibility of this approach by reaching an overall accuracy of 82% when identifying a set of 14 events in an indoor environment. Obtained results encourage practitioners to keep working in this direction, and allow health care providers to remotely track the status of their patients in real-time with non-invasive methods. The next experiments will be centered on performing the tests in a richer data set, both in number of samples and number of events, and to improve the overall accuracy of the system. Other feature extraction proposals will be tested (e.g., Gammatone Cepstral Coefficients, Gammatone Wavelet Cepstral Coefficients, etc.), and also other data mining algorithms will be used to perform the classification to improve its results (e.g., Deep Neural Networks, Gaussian Mixture Models, etc.).

Future work in terms of application will focus on two key points. The first is the analysis of the temporary patterns of the events that are already recognized in this proof-of-concept; the goal will be to know their duration and their occurrences during the day and night. These events will be temporarily monitored in order to reach conclusions regarding the patients behavior at home. The second key point of future work is to decide how many events and which ones the homeSound platform will detect. The processing of the information after the event recognition will be matched with the meaning of the occurrence or non-occurrence of the event, as well as its duration and the number of occurrences. The time dimension of the information study increases the control and monitoring possibilities in the house: (i) absence of noise at key moments; (ii) presence of a lot of people; (iii) phone or bell ringing insistently; (iv) night activity; (v) television operating for hours, as well as classic surveillance events such as (vi) any fall or door closed violently and (vii) glass broken. The combination of the event detection algorithm together with the time study of the events detected will led us to an AAL system capable to set an alarm just after a crisis situation meanwhile it tracks several acoustic events to monitor the patients behaviour at home. This information is relevant for care-providers since it makes them aware of the patients daily routines and also warns them of abnormal situations at home in certain moments.

## Figures and Tables

**Table 1:** Computational cost of the audio event detection algorithm proposed in this work.

Algorithm	Computational Cost	Floating Point Operations
Hamming Windowing	*M*	1.440
FFT	$N*log_{2}\left(N\right)$	22.528
${\left(abs\right)}^{2}$	N/2	10.24
Filter Bank	N/2*R	49.152
Logarithm	*R*	48
DCT	R*D	624
Subspace transformation	D*X	273
SVM${}_{test}$	2*X+((E*(E−1)/2)*2*X)	1840

**Table 2:** Confusion matrix of the hierarchical classifier. Predicted classes are on the columns and actual classes are on the rows.

	Falling down	Knife Slicing	Screaming	Raining	Printing	Talking	Frying	Filling Water	Knocking a Door	Dog Barking	Car Horn	Breaking Glass	Baby Crying	Water Boiling
Falling down	62.07	2.87	1.72	7.47	6.32	2.23	0.00	4.02	6.90	1.15	3.85	0.17	0.00	1.23
Knife slicing	3.67	67.46	1.04	7.29	0.00	3.15	2.18	1.04	4.68	4.78	0.00	0.15	1.43	3.03
Screaming	2.42	2.08	91.72	0.00	1.29	0.11	0.18	1.33	0.13	0.48	0.13	0.00	0.00	0.13
Raining	1.11	0.05	0.12	96.33	0.79	0.19	0.23	0.17	0.23	0.10	0.04	0.03	0.17	0.45
Printing	9.61	0.00	0.67	0.00	84.67	0.22	0.16	0.64	1.33	0.10	0.95	0.77	0.13	0.75
Talking	2.97	3.12	1.91	1.51	2.14	78.14	0.92	1.16	2.79	0.62	2.63	1.65	0.11	0.33
Frying	3.89	4.08	2.76	1.97	0.18	0.12	83.19	0.03	0.74	0.48	0.91	0.61	0.86	0.18
Filling water	2.08	0.94	2.86	0.00	1.96	0.84	0.39	73.73	0.00	2.6	1.96	0.41	5.52	6.71
Knocking a door	1.41	0.75	1.96	2.71	0.96	0.93	0.06	0.00	88.63	0.04	0.00	0.16	0.44	1.96
Dog barking	2.19	0.82	3.19	0.16	2.92	1.79	0.52	0.14	0.64	87.15	0.08	0.13	0.17	0.10
Car horn	1.11	5.95	1.33	0.00	0.47	0.00	0.29	1.19	1.19	0.07	79.05	0.31	0.71	8.33
Breaking glass	0.21	1.74	0.91	1.67	0.91	0.74	0.18	0.32	0.15	0.13	0.09	92.17	0.67	0.11
Baby crying	3.08	0.00	4.14	1.29	3.80	1.83	2.48	0.11	0.06	0.34	0.02	0.19	82.54	0.12
Water boiling	1.08	2.15	0.69	0.00	2.08	0.38	1.71	0.33	1.39	1.86	2.08	3.25	1.83	81.17

## Abbreviations

The following abbreviations are used in this manuscript:

AAD	Acoustic Activity Detection
AAL	Ambient Assisted Living
ADC	Analog to Digital Converter
ADL	Activities Daily Living
ARM	Advanced RISC Machines
BSP	Board Support Package
CPU	Central Processing Unit
CUDA	Compute Unified Device Architecture
DCT	Discrete Cosine Transform
DFT	Discrete Fourier Transform
DNN	Deep Neural Networks
DTW	Dynamic Time Warping
HPC	High Performance Computing
FFT	Fast Fourier Transform
FPGA	Field Programmable Gate Array
GFLOP	Giga-Floating Point Operations
GMM	Gaussian Mixture Models
GPU	Graphic Processing Unit
GP-GPU	General Purpose Graphics Processing Unit
GRITS	Grup de Recerca en Internet Technologies & Storage
GTM	Grup de Recerca en Tecnologies Mèdia
HMM	Hidden Markov Model
IoT	Internet of Thing
MEC	Mobile Edge Computing
MFCC	Mel Frequency Cepstral Coefficient
RFID	Radio Frequency Identification
SIMT	Single Instruction Multiple Thread
SoC	System on Chip
SNR	Signal to Noise Ratio
SVM	Support Vector Machine
t-SNE	t-Distributed Stochastic Neighbor Embedding
WASN	Wireless Acoustic Sensor Network
WFS	Wave Field Synthesis
WSN	Wireless Sensor Network
